# Dispersant Effects on Single-Walled Carbon Nanotube Antibacterial Activity

**DOI:** 10.3390/molecules27051606

**Published:** 2022-02-28

**Authors:** Matthew M. Noor, Alinne L. R. Santana-Pereira, Mark R. Liles, Virginia A. Davis

**Affiliations:** 1 Department of Chemical Engineering, Auburn University, Auburn, AL 36849, USA; noormatthew@gmail.com; 2 Department of Biological Sciences, Auburn University, Auburn, AL 36849, USA; alp0051@auburn.edu (A.L.R.S.-P.); lilesma@auburn.edu (M.R.L.)

**Keywords:** antibacterial, carbon nanotube, surfactant

## Abstract

There is significant interest in understanding whether nanomaterials with outstanding mechanical or electrical properties also possess antibacterial properties. However, assessment of antibacterial activity is a complex problem at the interface of chemistry and microbiology. Results can be affected by many factors including nanomaterial size, surface chemistry, concentration, and the dispersion media. The difficulty of dispersing nanomaterials such as single-walled carbon nanotubes (SWNTs) has resulted in many studies being conducted in the presence of dispersion aides which may themselves contribute to bacterial stress. The recent discovery that a standard microbial growth media, tryptic soy broth (TSB), is an effective SWNT dispersant provides a new opportunity to investigate the potential antibacterial activity of SWNTs using dispersants that range from antibacterial to growth-supporting. The five dispersants chosen for this work were Sodium dodecyl sulfate (SDS), pluronic, lysozyme, DNA, and tryptic soy broth. *Staphylococcus aureus* and *Salmonella enterica* were used as the model Gram-positive and Gram-negative bacteria. Activity was measured in terms of colony forming unit (CFU) and optical density measurements. None of the systems exhibited activity against *Salmonella*. SDS was fatal to *Staph. aureus* regardless of the presence of SWNTs. The activity of pluronic and lysozyme against *Staph. aureus* was enhanced by the presence of SWNTs. In contrast, the DNA and TSB dispersions did not have any activity regardless of the presence of SWNTs. These results highlight that the purported antibacterial activity of SWNTs may only be effective against bacteria that are sensitized by the dispersant and suggests the need for additional research on the mechanisms by which SWNT-dispersant interactions can result in antibacterial activity.

## 1. Introduction

Globally drug-resistant bacteria cause an estimated 700,000 deaths per year; this is predicted to rise to 10 million by 2050 [1]. Nanomaterials’ physical size and high specific surface area often make them of interest for the development of new antibacterial compounds and structures. However, assessing antibacterial activity is a complex task where the experimental protocol, type of bacteria, and details of nanomaterial size, surface chemistry, and dispersion media can all affect results. This complexity combined with the tendency to simply state that a nanomaterial is (or is not) antibacterial without providing additional study details can result in significant controversy in the literature. For example, nanosilver is widely accepted as being antibacterial and several commercial applications tout its antibacterial properties. However, as pointed out by Duval et al. (2019), the differences in research protocols make it challenging to directly compare studies and assess the reasons for discrepancies [2]. Similar challenges are seen with carbon nanomaterials such as graphene oxide [3,4] and single-walled carbon nanotubes (SWNTs). As described by Palmieri et al., studies of potential graphene oxide activity have yielded conflicting results due to issues such as variations in size and test protocol [5]. Studies of the potential activity of SWNTs are even more challenging. Their perfect sp^2^ hybridized structure results in attractive van der Waals interactions on the order of 20–40 k_B_T per nm of length [6]. Since SWNTs are typically on the order of one micron long, the attractions between them are stronger than a covalent bond. As a result, they are notoriously difficult to disperse in most solvents. Dispersion in aqueous media can only be achieved by either oxidizing them to create carboxylic and hydroxyl groups on their surfaces or using dispersion aides that non-covalently adsorb on their sidewalls. This dispersion challenge as well as their structural polydispersity has complicated efforts to assess whether they have antibacterial activity. The first approach, oxidation, reduces SWNTs’ outstanding mechanical, electrical, and thermal properties. Moreover, the specifics of the oxidation protocol result in different surface groups which have been shown to affect antibacterial activity [7]. The inclusion of dispersion aides in studies of pristine SWNTs introduces a confounding factor since many common SWNT dispersion aides such as sodium dodecyl sulfate (SDS), which is widely used in soap, are known to have antibacterial properties.

Both non-physiological media such as deionized water and dispersion aides such as SDS can stress bacteria and affect their growth. Previous studies of SWNTs’ potential antibacterial properties have examined the effects of purity, chirality distribution, diameter, length, and dispersion state [7,8,9,10,11,12,13,14]. Many of these studies have concluded that SWNT possess antibacterial properties and attributed their findings to a range of potential mechanisms including metabolic disruption or inhibition [15], oxidative stresses [8,15,16], and physical piercing damage to the cell membrane [8,17,18]. However, very few studies have explored the potential for SWNTs’ perceived activity to be the result of synergistic interactions with the dispersant. A notable exception is Arias and Yang (2009) who found that the activity of oxidized SWNTs against *Salmonella* was both surface-chemistry- and media-dependent [7]. They found significant, surface-chemistry-dependent activity in deionized water and 0.9% sodium chloride solution. However, they did not observe activity in either phosphate buffer solution (PBS) or brain heart infusion (BHI) broth, which are both physiological media. The pursuit of similar studies has been hindered for pristine SWNT because of the limited range of available dispersants.

The relatively recent finding by Sloan et al. that a standard microbiological growth media, tryptic soy broth, can disperse SWNTs as individuals and small bundles [19] has created a new opportunity for the direct comparison of SWNT activity in media ranging from mildly bactericidal to growth-supporting. Therefore, in contrast to the existing literature, this work uses the same protocols to explore the activity of SWNTs in both the SDS and pluronic commonly used as pristine SWNT dispersants and the biological dispersants DNA, lysozyme, and tryptic soy broth (TSB). The effects of the dispersions on *Staphylococcus aureus* (Gram-positive) and *Salmonella enterica* (Gram-negative) were compared through colony forming unit (CFU) counts and comparisons of bacterial growth curves optical density measurements at 600 nm (OD_600_). The results highlight the importance of considering synergistic interactions when assessing antibacterial activity.

## 2. Results

**Figure 1:**
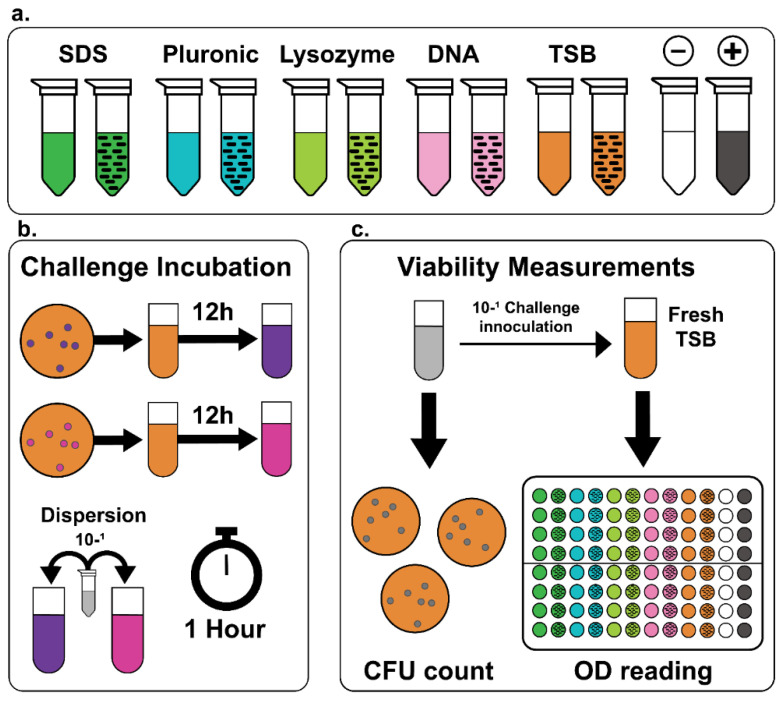
Experimental design of the SWNT antimicrobial challenges. (**a**) Dispersions used in the experiment. Cultures were challenged against the dispersant and the dispersant with SWNTs. (**b**) Challenge incubation. Overnight growth cultures of *Salmonella* and *Staph. aureus* were diluted in fresh TSB to OD_600_ = 0.5 and incubated with the challenging solution for one hour. (**c**) The challenge incubations were platted for CFU counting and inoculated into fresh TSB for growth curve construction.

The effects of different dispersants on SWNTs’ activity toward bacteria were directly compared using CFU and OD_600_ measurements. CFU counts provide a way to rapidly assess activity by exploring the number of colony units after incubation with a material, while OD_600_ can be used to evaluate the effects of a treatment (such as the inclusion of carbon nanotubes) on a growth curve. The overall methodology is shown in Figure 1; additional details about the test method and statistical analysis are provided in the Section 3.

*Staphylococcus aureus* and *Salmonella enterica* were chosen as model Gram-positive and Gram-negative bacteria due to their clinical relevance and use in other studies. The dispersants were chosen based on a combination of their ability to disperse pristine SWNT and the desire to include dispersants with a range of bacterial activity. SDS, which is included in many soaps, and lysozyme, which is used in some mouthwashes, are known to be active against Gram-positive bacteria. Pluronic was expected to have little bactericidal activity and DNA no activity. TSB, which has not previously been used in studies of SWNT bacterial activity, is a standard growth media that provides nutrients to support bacterial growth.

**Figure 2:**
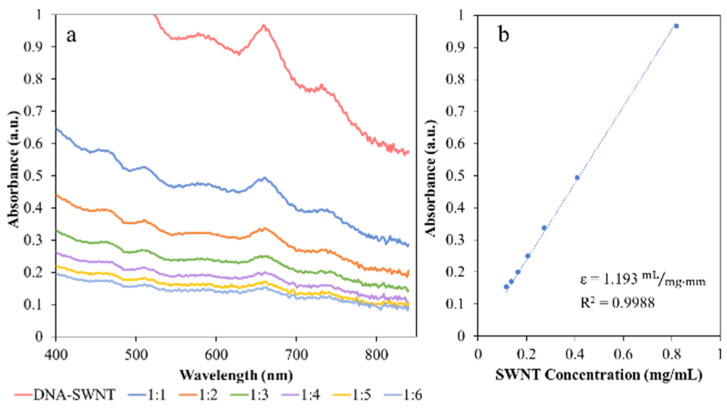
Example UV-Vis spectra of a serial dilution using the NanoDrop200c and resulting Beer-Lambert plots used to obtain the extinction coefficients in Table 1 and concentrations based on the Beer Lambert Law (*A = εlc*). The initial dispersion concentrations prior to dilution were based on drying dispersions in TGA pans. (**a**) UV-vis spectra of successive dilutions of DNA-SWNT dispersions and (**b**) Beer-Lambert plot for DNA-SWNT showing the extinction coefficient corresponding to the slope.

Even with dispersion aides such as the ones used in this study, pristine individual and small bundles of SWNT can only be dispersed in aqueous media at concentrations on the order of 1 mg/mL. The specific concentration that can be achieved is the result of thermodynamic interactions between SWNT, the dispersant, and the solvent (water). Increasing the SWNT concentration above this amount can result in aggregation; even increasing the relative concentration of the dispersant is problematic because of potential depletion interactions [20]. Kinetically stable dispersions of SWNT following literature methods were developed from detailed studies of SWNT dispersion behavior. As described in Section 3, this consisted of tip sonication followed by centrifugation to achieve supernatants containing SWNT dispersion aide adducts. UV-vis spectra were acquired for successive dilution of the supernatants and were used to obtain Beer-Lambert plots According to the Beer-Lambert law A=εlc where *ε* is the extinction coefficient, *A* is the absorbance at a specified wavelength, l is the path length, and c is the concentration of the dispersion. The absorbance at 660 nm was chosen for determination of the extinction coefficient because this was the location of a strong van Hove singularity peak, an indicator of individual SWNT dispersion. Figure 2 shows representative spectra for the DNA-SWNT dilution series as well as the corresponding Beer-Lambert plot where the slope corresponds to the extinction coefficient. Table 1 provides the corresponding extinction coefficients, which were used for determining the concentration of additional samples.

As expected, the SWNT concentration in each of the initial supernatants varied with the dispersant type (Table 2). SDS is one of the most commonly used SWNT dispersants having the highest concentration of 1.67 mg/L followed by DNA, the best biological SWNT dispersant having a concentration of 0.72 mg/mL. Pluronic and TSB had intermediate concentrations of 0.58 and 0.45 mg/mL, respectively, while lysozyme resulted in the lowest concentration, 0.23 mg/mL. These values were similar to literature values [10,19,20,21,22,23] with the exception of SDS, which has a higher final dispersion concentration than reported in the literature [11,24]. Since SWNTs are polydisperse in length, and supernatants can contain both individuals and small bundles, atomic force microscopy (AFM) was used to measure the sizes of the SWNT-dispersant adducts present in each sample. Representative images are shown in the supporting information (Figure S1). To allow for diameter polydispersity and the size of the attached dispersant, entities with measured heights lower than 3 nm were considered to be individual SWNT while those with larger heights (diameters) were considered bundles. As shown in Table 2, the majority of SWNT in the SDS sample existed as bundles, which may explain the higher-than- expected total concentration. The other samples were predominantly individuals. Interestingly, TSB (which was only recently identified as a good SWNT dispersant [19]) had the highest individual-to-bundle ratio. Length measurements showed the expected polydispersity, with all samples having an average length of approximately 200 nm due to the use of ultrasonication.

The growth of *Staph. aureus* and *Salmonella* after a one-hour incubation with each SWNT dispersant (Figure 1) was determined using CFU counts with water as a control. Details of the protocol, including statistical analysis, are described in Section 3. To compare the effect of SWNT concentration on bacterial viability, incubations were performed using both the full supernatant concentration and samples normalized to the lowest SWNT concentration (0.227 mg/mL) via dilution with DI water. Determining CFU counts allowed the assessment of viable bacterial cells while avoiding SWNT interference in fluorescence- or absorbance-based bacterial viability assays.

**Figure 3:**
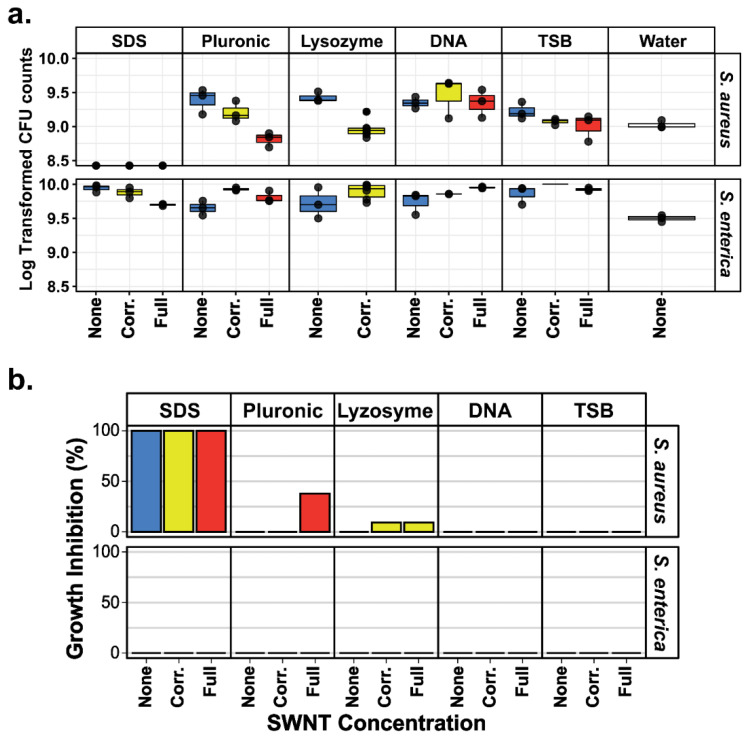
Cell viability measured by CFU counts. (**a**). CFU counts in triplicate of the challenges for *Staph. aureus* and *Salmonella*. Log 10 transform was used to coerce the data into a more normal distribution and facilitate visualization. (**b**). Antimicrobial activity of the dispersants with different SWNT concentrations. Antimicrobial activity measured as cell viability loss in relation to a water challenge. None: No SWNTs; Corr.: SWNT concentration corrected to 0.23 mg/mL for comparison across all dispersants; Full: Full-strength SWNT concentrations as shown in Table 2.

As shown in Figure 3, the results for *Staph. aureus* were sample-dependent. Gram-positive bacteria have a thick cell wall comprised of many layers of peptidoglycan that give the cell structural support. The dispersants that are known to act directly on the bacterial cell wall (SDS, lysozyme, and pluronic) and destabilize the peptidoglycan structure resulted in inhibition either alone or in the presence of SWNT. *Staph. aureus* was very susceptible to the surfactant SDS regardless of the presence of SWNT (*p* = 0.023). Interestingly, the mild and non-cytotoxic pluronic had no effect on *Staph. aureus* viability except at the higher (full) SWNT concentrations (*p* = 0.0201). While a slight reduction in *Staph. aureus* viability was observed when cells were incubated with both lysozyme and SWNTs, this result was not statistically significant (*p* = 0.083) (Figure 3). Notably, *Staph. aureus* was unaffected by SWNTs, regardless of their concentration, when they were dispersed in TSB or DNA, both of which are biologically compatible dispersants. These findings are in agreement with Arias and Yang’s finding that oxidized SWNTs’ activity depended on their surface chemistry and dispersant [7].

In contrast to *Staph. aureus,* the CFU counts of gram-negative *Salmonella* were not affected by any of the dispersants or the presence of SWNT treatments. Gram-negative bacteria like *Salmonella* (which is affiliated with gamma-proteobacteria such as *Escherichia coli*) have an inner and outer membrane between which is the periplasm that includes a thinner peptidoglycan layer. Because of their double membrane structure, Gram-negative bacteria are less vulnerable to agents such as lysozyme and show higher surfactant resistance [25,26].

In addition to measuring bacterial growth inhibition via CFUs after a 1 h exposure to SWNT-dispersant combinations, the effect of the treatments was also assessed by monitoring the OD_600_ for 24 h (see Section 3). Albeit indirect, determining bacterial growth using OD_600_ allows the estimation of different metrics of growth such as initial population, rate of growth, doubling time, environment carrying capacity, and overall growth comparisons, and their differences from the controls. Since both SWNTs and bacteria absorb light at 600 nm, the bacterial suspensions were first exposed to SWNTs for 1 h and then diluted 10-fold in TSB and monitored for growth to remove absorbance interference.

**Figure 4:**
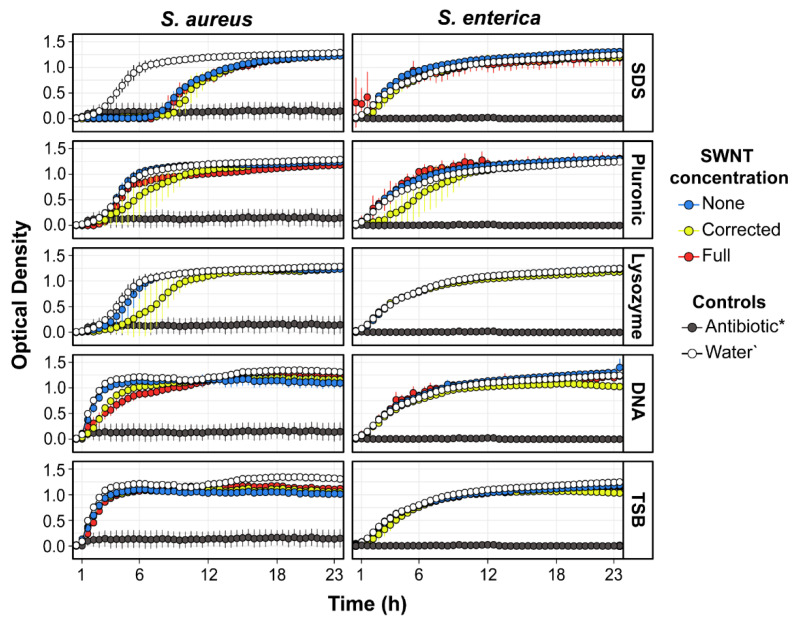
Growth curves of inoculums of *Staph. aureus* and *Salmonella* after challenge with the 17 treatments TSB medium. Growth measured as optical density at 600 nm wavelength every 30 min for 24 h. Longer lag phases indicate that cells were sustaining damage and/or under increased stress. None: no SWNTs; Corrected: SWNT concentration corrected to 0.227 mg/mL for comparison across all dispersants; Full: full-strength SWNT concentrations as shown in Table 2. Antibiotic*: Chloramphenicol for *Salmonella* and vancomycin for *Staph. aureus*.

The growth curves from the OD_600_ studies are shown in Figure 4, and the parameters extracted from the data are shown in Figure S2. In order to compare the overall growth of the inoculums, the area under the curve was calculated by integration, which summarizes the contributions of the carrying capacity, initial population, and growth rate into a single value [27]. Similar to the CFU results, none of the samples affected the *Salmonella* growth or growth rate (*p* < 0.05). Treatment with SDS and DNA with the corrected SWNT concentrations did have significant effects on initial population (*p* = 0.017 and *p* = 0.031, respectively). However, these changes did not impair *Salmonella* overall growth and thus did not result in inhibition.

All treatments with SDS, including SDS alone, produced a sharp reduction in the overall growth of *Staph. aureus* (*p* < 0.009). Inoculums from these treatments had similar growth rates; however, they took significantly longer to achieve their maximum growth rate (*p* = 0.002), emphasized by their extended lag phase, which indicates sustained damage or stress [28]. A similar, albeit less pronounced, effect was observed in the treatments with pluronic and lysozyme with 0.23 mg/mL of SWNT where there was a significant reduction in growth rate (*p* = 0.02 and *p* = 0.041, respectively) and a longer lag phase for lysozyme. This resulted in a reduction in overall growth in both cases. Additionally, treatment with full-strength SWNT dispersions in pluronic also led to decreased overall growth, while the pluronic and lysozyme alone did not. These observations are in agreement with the CFU results.

Treatments with DNA plus SWNTs had significantly slower growth rates (*p* < 0.0006), with higher generation times (*p* < 0.03). These differences led to a significant decrease in overall growth for treatment with corrected SWNT concentration dispersed in DNA (*p* = 0.002), but not for full the strength SWNT concentration. Interestingly, all TSB treatments, including the samples without SWNT, had lower overall growth values relative to the water control, including TSB alone (*p* < 0.005). These incubations had a significantly lower carrying capacity (*p* < 0.036), which caps the possible overall growth. Inspecting the growth curves (Figure 3) did not indicate inhibition; there was no extension of lag phase. The maximum growth rate was achieved at similar times, and the stationary phase was simply reached at a lower optical density than the control.

Together the CFU and OD_600_ results suggest that the impact of SWNT on bacteria is strongly influenced by whether the bacteria are stressed by the dispersant or other sensitizing factors. No significant activity was observed for *Salmonella*, and SWNT only exhibited antibacterial activity against *Staph. aureus* in the presence of cell wall sensitizing agents. These findings are consistent with the previously described work by Arias and Yang [7], where inhibition was not seen in PBS and BHI but was observed in DI water and 0.9% saline solution. It is noted that dispersants like water can seem innocuous [7,8,9], but they do not contain the nutrients needed to sustain healthy bacteria. In addition, hypotonic media can be a sensitizing factor and facilitate cell wall disruption by increasing osmotic stress. Moreover, bacterial resistance against surfactants requires increased nutrient uptake [25,29]. Consequently, exposing bacterial cells to SWNTs in a medium devoid of nutrients might exacerbate loss of viability by denying living cells the resources needed to withstand stress.

The idea of synergistic effects in bacterial activity is not new. Indeed, a similar synergistic effect between surfactants and traditional antibiotics has been long described in the literature [30]. Synergistic effects have also been explored in the context of food, cosmetics, and other applications. For example, SDS has been used to increase organic acids’ efficacy against *Salmonella* in poultry [31]. Similarly, essential oils and surfactants have been used to increase the effectiveness of conventional biocides in cosmetic formulations [32]. However, the emerging nanomaterial literature has not yet explored the potential for such synergistic interactions in detail. The challenges of dispersing pristine SWNTs in aqueous media as well as their polydispersity complicate obtaining dispersions where the SWNTs have the same concentration, length, and diameter distribution. Moreover, dispersion of pristine SWNTs is largely achieved by adsorption of the dispersant on SWNT sidewalls; different dispersants result in different coverage of the SWNT sidewall surface and different effective surface chemistries and diameters. In some studies, differences in activity in different media were attributed to differences in SWNT dispersion state and not to the media itself. For example, Liu, et al. showed that effectiveness against Gram-negative *E. coli*, *P. aeruginosa*, and Gram-positive *B. subtilis* was dispersant-dependent, with the greatest effect observed for the Gram-positive bacteria. Specifically, they found that the activity of a mixture of the surfactant Tween-20 and 0.9% NaCl was slightly greater than that of 0.9% NaCl alone. In addition, the activity of SWNT dispersed in sodium cholate (which has inherent antibacterial properties) was greater than for sodium cholate alone and the other dispersions [18]. They remarked on the potential for sodium cholate to reduce the structural integrity of the cell membrane but did not study it in as much depth as the other dispersants. They concluded that the difference between 0.9% NaCl and 0.9% NaCl+ Tween-20 was due to the presence of SWNT bundles in the absence of Tween-20, but they did not consider the possibility that the Tween-20 could have sensitized the bacteria to damage by SWNT. They did, however, conclude that SWNTs were more effective against softer bacteria [18,33], which would be in agreement with our studies where the activity was related to the dispersants’ capacity to cause bacterial stress or damage but was not correlated to individual-to-bundle ratio. Future investigation of cell wall structure after exposure to dispersants and additional studies in TSB or other growth media are required to further understand the role of media-induced bacteria sensitization to SWNT. Such studies should include detailed investigation of mechanisms of action in order to enable the design of SWNT dispersions with a desired level of antibacterial properties for a given application.

## 3. Materials and Methods

### 3.1. SWNT Dispersion Preparations

SWNTs (CG300L35) for this research were purchased from CHASM (Norman, OK, USA) and used as received. Lysozyme from hen egg white, double-stranded DNA sodium salt from salmon testes, and pluronic F108 were purchased from Sigma-Aldrich (St. Louis, MO, USA). Sodium dodecyl sulfate (SDS) was purchased from Fisher Scientific (Hampton, NH, USA). Finally, BD Bacto (Franklin Lakes, NJ, USA) TSB mixture was prepared according to vendor instructions.

The dispersants and SWNT were dispersed using initial concentrations and sonication protocols from the literature (Table 3). After sonication, each sample was centrifuged at 17,000× *g* for 3 h to remove large SWNT bundles and aggregates. The supernatant was collected and used for the experiments. For normalized concentrations, the SWNT dispersions were diluted with their respective dispersant controls to a corrected concentration of 0.227 mg/mL.

### 3.2. SWNT Dispersion Chracterization

A Thermo Scientific (Waltham, MA, USA) NanoDrop 2000c UV-visible spectrophotometer was used to measure absorbance and subsequently determine concentration by the Beer-Lambert equation. Samples were diluted with varying amounts of DI water to maintain absorbance values lower than 1.0 in the range of the observed van Hove peaks for this purpose.

Atomic force microscopy (AFM) was performed with a Pacific Nanotechnology (Berkley, CA, USA) Nano-R SPM. AFM samples were prepared by dropping a single drop of 50 ppm (0.005 vol%) dispersion, diluted by DI water, onto freshly cleaved mica sheets. The sample was allowed to rest on the surface for 10 min, then rinsed with DI water and vacuum dried at 50 °C for at least 12 h. The AFM was operated in non-contact mode at a resolution of 1024 and a scan rate of 0.25 s^−1^. The diameters of over 100 entities were measured for each sample, with some samples requiring multiple scans. Entities with measured heights greater than 3 nm were considered to be bundled SWNTs; lower heights were considered to be individual SWNTs.

### 3.3. Bacterial Strains and Cultivation

Bacterial strains of Gram-positive and Gram-negative species were used in this study. For a representative Gram-positive bacterium, *Staphylococcus aureus* Xen29 (Perkin Elmer, Johns Creek, GA, USA) was selected, and for a Gram-negative bacterium *Salmonella enterica* subsp. *enterica* (ATCC: 14028) was selected. Each bacterial species was grown from a glycerol stock onto a Tryptic Soy Agar (TSA) medium to obtain isolated colonies and incubated at 37 °C overnight. An isolated colony from each culture was used to inoculate 2 mL of tryptic soy broth (TSB), and the culture tubes were incubated at 37 °C with shaking at 200 rpm overnight.

### 3.4. Bacterial Culture Challenges and Viability Measurements

The overnight broth cultures were normalized to an optical density at 600 nm (OD_600_) of 0.5 by dilution with TSB. Measurements of turbidity at OD_600_ were taken using a spectrophotometer. Then 360 µL of the normalized bacterial suspensions were inoculated with 40 µL of a specific treatment group containing either sonicated dispersants without SWNT (pluronic, SDS, lysozyme, DNA, or TSB), SWNT dispersions (in pluronic, SDS, lysozyme, DNA, or TSB), or SWNT dispersions with their concentration corrected (normalized) to that of the lysozyme dispersion (Figure 1). The antibiotic vancomycin was used as a positive control to inhibit *Staph. aureus* growth; chloramphenicol was used as a positive control to inhibit *Salmonella* growth; and sterile MilliQ water was used as a negative control, resulting in a total of 17 treatments per bacterial strain. Each of the bacterial treatment group suspensions was incubated for 1 h at 37 °C with shaking at 200 rpm.

An aliquot of each treatment group sample was then inoculated at a 10^−1^ dilution into fresh TSB and transferred to a clear 96-well plate in quadruplicate, with 200 µL per well. The plate containing all the treatment groups was incubated for 24 h with shaking at 37 °C in a microtiter plate reader and the OD_600_ was measured every 30 min to determine growth curves. A second run of the experiment was performed for the TSB and DNA treatments after the inoculums failed in the first 24 h incubation attempt due to excessive dehydration at the border of the 96-well plate. The OD_600_ measurements were corrected for media and SWNTs absorbance by subtracting the values at time zero from all consecutive measurements from each well. Mean and standard deviation of the replicates were used to build the reported growth curves.

Another aliquot of each sample was serially diluted and plated onto TSA plates to determine the number of CFUs per mL in triplicate. The plates were incubated for 24 h, and the dilutions yielding roughly 30–300 colonies were picked for CFU determination. Reported inhibition was calculated as the difference between mean CFU count between challenges and negative control treatment (water).

### 3.5. Statistical Analysis

Growth curves from all individual replicates were analyzed using the R package Growthcurver [27]. Briefly, a logistic model was fitted to each growth curve from which the carrying capacity, initial population, growth rate, maximum growth rate, and doubling times were calculated. Additionally, the area under the curve was calculated by integration, which summarizes the contributions of carrying capacity, initial population, and growth rate in one parameter. Then replicates of each of these parameters were averaged and used for comparisons between treatments. For each of the dispersant groups, each parameter was compared by a Welch’s ANOVA test to evaluate if there were differences between the means of the treatments: dispersant alone (None), dispersant + SWNTs with corrected concentration (Corr), dispersant + SWNTs full strength (Full), and water. Following a positive Welch’s ANOVA, Games-Howell’s tests with confidence intervals of 0.95 were performed to identify the treatments that were significantly different from each other. These tests were chosen over the traditional ANOVA and Tukey’s tests because the data violated the assumption of homogeneity of variance.

In the case of CFU counts, non-parametric methods were preferred since the data were not normally distributed. For each dispersant, the Kruskal-Wallis test (analogous to ANOVA) was first conducted to evaluate if there were differences between the means of the treatments: dispersant, dispersant + SWNTs with corrected concentration, dispersant + SWNTs full strength, and water. Following a positive Kruskal-Wallis, Dunn tests (analogous to Tukey’s test) were performed, in the same manner as described above. Again, inhibition was considered as a significant difference between a treatment and the water control. All analyses and plots were performed and drawn, respectively, in R-studio. Charts were drawn in Adobe Illustrator.

## 4. Conclusions

Our results highlight that attributing the presence or absence of SWNT dispersion antibacterial activity to the SWNTs alone can be an oversimplification. Activity measurements can be strongly affected by synergistic interactions between SWNTs and dispersants which exert stress on the cells. In this study, SWNTs were not able to kill healthy *Staph. aureus* cells in the absence of dispersants that are known to induce bacterial stress. None of the samples were active against *Salmonella*; this is attributed to Gram-negative bacteria being less vulnerable to cell wall stress. The discovery that SWNTs can be dispersed in TSB provides an opportunity for future research that includes both this growth media and more commonly used dispersants to further decouple SWNT and dispersant effects. Such future studies will extend understanding of how combinations of SWNTs and dispersants interact with microbial cells and the combined mechanism(s) by which they can drive cell lysis. Such studies will also need to take into consideration the bacterial type and environment, the SWNT type and dispersion state, and other potential confounding factors. They should also include careful study of bacteria under different sensitization factors using both standard microbiological methods and methods such as atomic force microscopy. While this study was limited to pristine SWNTs, it is hoped that these results will also result in consideration of dispersants and other potential physiological stressors in studies of other nanomaterials including graphenes and MXenes.

## Figures and Tables

**Table 1:** Extinction coefficients calculated from UV-vis spectra Beer-Lambert plots.

Dispersion	Extinction Coefficient SWNT (mL/mg mm)
SWNT-LSZ	0.759
SWNT-DNA	1.193
SWNT-Pluronic	14.53
SWNT-SDS	5.422
SWNT-TSB	0.200

**Table 2:** SWNT concentrations, individual-to-bundled SWNT ratio, and average length of individual SWNT for each dispersion.

Sample	[SWNT] mg/mL	Individual to Bundle Ratio	Avg. Individual Length (nm)
SWNT-LSZ	0.23	3.2	190 ± 49
SWNT-DNA	0.72	5.7	220 ± 52
SWNT-Pluronic	0.58	6.7	170 ± 47
SWNT-SDS	1.7	0.4	170 ± 35
SWNT-TSB	0.45	9.0	230 ± 60

**Table 3:** Initial concentrations and sonication protocols from each dispersion based on the literature.

Dispersant	[Dispersant] (WT %)	[SWNT] (WT %)	Sonication Procedure		
			Time (min)	Amplitude (%)	Pulse (5 s on, 2 s off)
SDS [11,24]	1	0.2	60	60	no
Pluronic [21,22]	2	0.2	60	60	no
TSB [19]	as prepared	0.1	30	60	yes
Lysozyme [20]	0.5	0.1	30	60	yes
DNA [10,23]	0.75	0.1	30	50	no

## Data Availability

Data requests should be sent to the corresponding author.

## Supplementary Materials

The following supporting information can be downloaded online: Figure S1: AFM images; Figure S2: Growth parameters calculated from the fitted logistic model

## Sample Availability

Samples of the compounds are not available from the authors.
