# Supplementary material for: Dispersant Effects on Single-Walled Carbon Nanotube Antibacterial Activity
Source: Molecules. 2022 Feb 28;27(5):1606. doi: 10.3390/molecules27051606 (PMC8911888; doi:10.3390/molecules27051606)
Supplement: Supplementary file 1 [file molecules-27-01606-s001.zip › molecules-1562344-supplementary.pdf]

Supporting Information

Dispersant effects on single-walled carbon nanotube antibacterial activity

Matthew M. Noor[1‡], Alinne L. R. Santana-Pereira[2‡], Mark R. Liles[2] and Virginia A. Davis[1*]
[1]Department of Chemical Engineering, Auburn University – Auburn University, AL, USA
[2]Department of Biological Sciences, Auburn University – Auburn University, AL, USA
‡Both authors contributed equally for this paper
* Correspondence should be addressed to davisva@auburn.edu

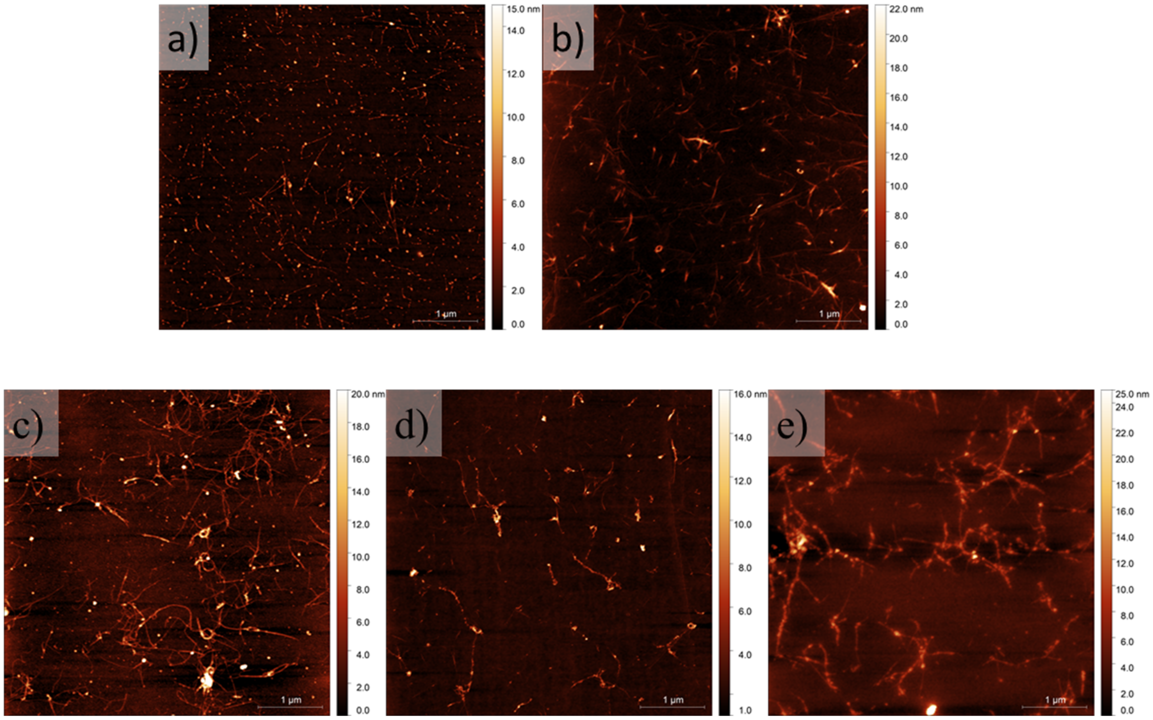

Figure S1. AFM scans of a) TSB-SWNT b) DNA-SWNT c) LSZ-SWNT, d) Pluronic-SWNT, and e) SDS-SWNT.

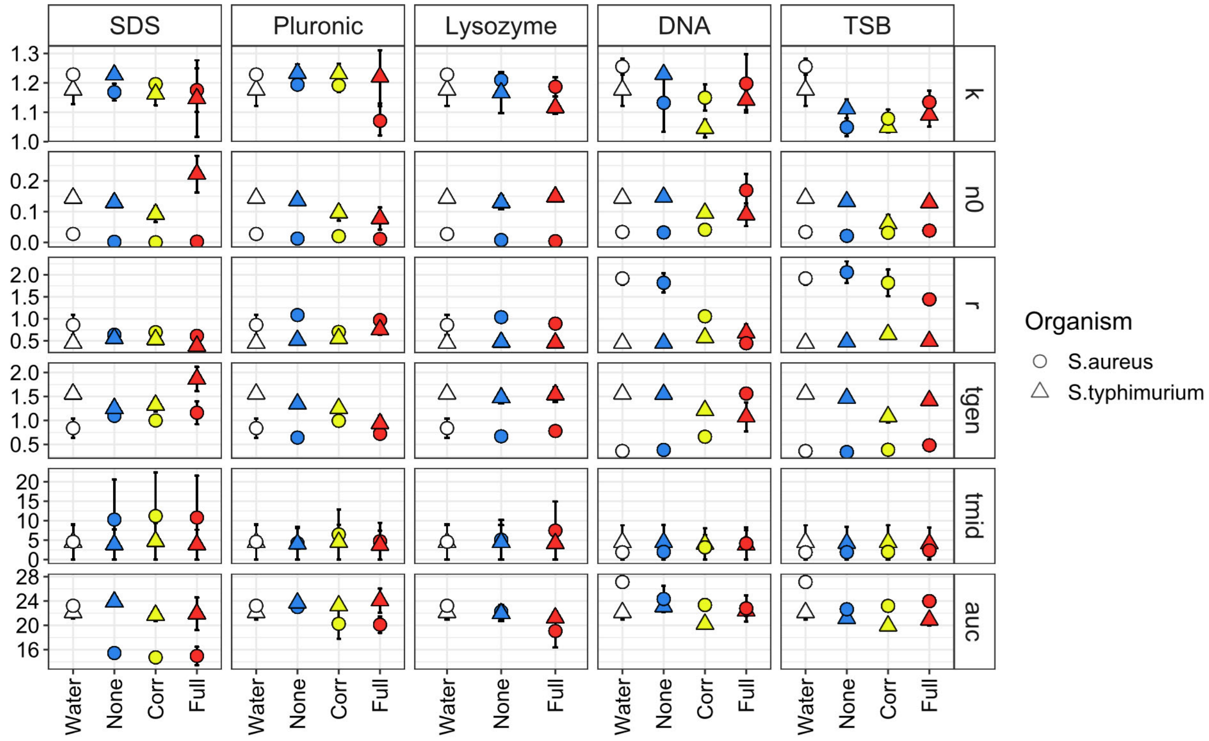

Figure S2. Growth parameters calculated from the fitted logistic model. **k**: Carrying capacity (expressed in OD values with arbitrary unit); **n0:** initial population at time 0 (expressed in OD values with arbitrary unit); **r**: growth rate (OD/hour); **tgen**: Generation time (hour), predicted time taken for population to double; **tmid**: maximum growth rate, time point where population reaches k/2 designated by the inflection point; **auc:** Area under the curve, integration of the logistic function summarizing k, n0 and r in one parameter that describes the curve. **None**: No SWNTs; **Corr**.: SWNT concentration corrected to 0.227 mg/mL for comparison across all dispersants; **Full**: Full strength SWNT concentrations as shown in **Table 1**.
